# Human Milk Oligosaccharides in Cord Blood Are Altered in Gestational Diabetes and Stimulate Feto-Placental Angiogenesis In Vitro

**DOI:** 10.3390/nu13124257

**Published:** 2021-11-26

**Authors:** Denise Hoch, Waltraud Brandl, Jasmin Strutz, Harald C. Köfeler, Mireille N. M. van Poppel, Lars Bode, Ursula Hiden, Gernot Desoye, Evelyn Jantscher-Krenn

**Affiliations:** 1 Department of Obstetrics and Gynecology, Medical University of Graz, 8036 Graz, Austria; denise1390@googlemail.com (D.H.); waltraud.brandl@medunigraz.at (W.B.); jasmin.strutz@medunigraz.at (J.S.); gernot.desoye@medunigraz.at (G.D.); 2 Institute of Biomedical Science, Carinthia University of Applied Science, 9020 Klagenfurt, Austria; 3 Core Facility Mass Spectrometry, Center for Medical Research, Medical University of Graz, 8010 Graz, Austria; harald.koefeler@medunigraz.at; 4 BioTechMed Graz, 8010 Graz, Austria; mireille.van-poppel@uni-graz.at; 5 Institute of Human Movement Science, Sport and Health, University of Graz, 8010 Graz, Austria; 6 Department of Pediatrics and Larsson-Rosenquist Foundation Mother-Milk-Infant Center of Research Excellence (LRF MoMI CoRE), University of California San Diego, La Jolla, CA 92093, USA; lbode@health.ucsd.edu

**Keywords:** pregnancy, gestational diabetes mellitus, human milk oligosaccharides, angiogenesis, cord blood, 3′-sialyllactose, placenta, tube formation, spheroid sprouting assay, fibrin tube formation assay

## Abstract

(1) Background: Human milk oligosaccharides (HMOs) are present in maternal serum during pregnancy and their composition is altered in gestational diabetes (GDM). HMOs are also in fetal cord blood and in contact with the feto-placental endothelium, potentially affecting its functions, such as angiogenesis. We hypothesized that cord blood HMOs are changed in GDM and contribute to increased feto-placental angiogenesis, hallmark of GDM. (2) Methods: Using HPLC, we quantified HMOs in cord blood of women with normal glucose tolerance (NGT, *n* = 25) or GDM (*n* = 26). We investigated in vitro angiogenesis using primary feto-placental endothelial cells (fpECs) from term placentas after healthy pregnancy (*n* = 10), in presence or absence of HMOs (100 µg/mL) isolated from human milk, 3′-sialyllactose (3′SL, 30 µg/mL) and lactose (glycan control) and determined network formation (Matrigel assay), proliferation (MTT assays), actin organization (F-actin staining), tube formation (fibrin tube formation assay) and sprouting (spheroid sprouting assay). (3) Results: 3′SL was higher in GDM cord blood. HMOs increased network formation, HMOs and 3’SL increased proliferation and F-actin staining. In fibrin assays, HMOs and 3’SL increased total tube length by 24% and 25% (*p* < 0.05), in spheroid assays, by 32% (*p* < 0.05) and 21% (*p* = 0.056), respectively. Lactose had no effect. (4) Conclusions: Our study suggests a novel role of HMOs in feto-placental angiogenesis and indicates a contribution of HMO composition to altered feto-placental vascularization in GDM.

## 1. Introduction

Human milk oligosaccharides (HMOs) are a complex blend of more than 150 structurally different highly bioactive glycans in human milk. HMOs consist of a lactose core and are further modified by fucosylation and/or sialylation. Biological functions of HMOs are tightly connected to their structural features [1]. In human milk, HMO composition and concentration vary between mothers, and within an individual over time. Part of the variation is explained by polymorphisms in the Secretor and Lewis genes, leading to varying activity in the encoded fucosyltransferases, FUT2 and FUT3, respectively, and the resulting distinct fucosylation patterns [2,3,4]. In addition to these genetic factors, emerging evidence suggests a contribution of environmental factors such as maternal nutritional and metabolic status to also influence concentration and composition of HMOs [5,6,7,8].

As highly bioactive molecules, HMOs are thought to provide various potential benefits to the breast-fed neonate. After ingestion by the infant, HMOs confer their effects not only locally as prebiotics and anti-adhesives, e.g., in the gut, but also systemically, after uptake into the blood, circulating the vasculature. Due to this known presence in the systemic circulation, HMOs have long been discussed as potential signaling molecules, interfering with lectin-glycan interactions, thereby inducing cell responses in different target cells such as leukocytes and endothelial cells [9,10,11]. Putative receptors for HMOs include a multitude of diverse carbohydrate binding lectin receptors, such as C-type lectins [12,13], galectins [14], siglecs [15], and selectins [11,16,17,18] as well as other surface molecules such as toll-like receptor 4 (TLR4) [10,19] or epidermal growth factor receptor (EGFR) [20].

Recent studies demonstrated that HMOs are present in the maternal circulation already during pregnancy [21,22]. This suggests that HMOs may not only target the breast-fed infant, but also have systemic effects in the pregnant woman and the unborn child. HMOs are detectable in urine and blood already in the first trimester of pregnancy [21,22,23,24,25]. Throughout pregnancy, maternal HMO concentration increases and HMO composition changes from a predominantly sialylated to a more fucosylated profile [21]. Metabolic parameters such as BMI, body fat mass or blood glucose are associated with concentrations of individual HMOs in maternal serum [21,22], with so far unknown effects on the pregnancy. In a cohort of overweight and obese women, we found higher serum 3′SL levels early in pregnancy in women who later developed gestational diabetes mellitus (GDM) [22]. GDM is a common pregnancy complication characterized mainly by glucose intolerance, hyperglycemia and hyperinsulinemia reflecting decreased maternal insulin sensitivity [26,27]. GDM alters fetal and feto-placental development resulting in an increased risk for placental abnormalities associated with perinatal morbidity, mortality [28,29,30,31] and long-term consequences for mother and offspring [29,31,32]. In a recent study, we demonstrated that HMO profiles in cord blood of newborn infants resembled those in maternal blood at delivery. This suggested maternal-to-fetal transfer, which we could confirm using an ex vivo perfusion model, in which individual HMOs crossed the placenta and accumulated in the fetal circuit [33]. It is unknown whether HMOs circulating the feto-placental vasculature are altered in GDM, and whether they directly impact the feto-placental unit, affecting endothelial functions in the placenta with implications for placental development and fetal vascular programming.

The placenta is the first fetal organ to develop and pivotal for fetal growth and development, as well as maintenance of pregnancy. Its vascular bed is the largest of the fetus, highly sensitive to growth factors, glucose, oxygen, nutrients, and vasoactive molecules in the fetal blood. Throughout gestation, feto-placental vasculature undergoes continuous expansion and remodeling, thus, relying on extensive angiogenesis. Angiogenesis involves consecutive distinct biological processes including cytoskeletal reorganization, migration, proliferation, sprouting and tube formation. These processes, and thus, placental development can be adapted by a deranged uterine environment as seen in maternal metabolic disorders such as pre-existing diabetes or GDM. In these pregnancies, placentas are structurally altered including changes in the vasculature [34,35,36,37]. These encompass increased capillary diameter and hypervascularization due to more vascular branching [38,39,40], collectively facilitating oxygen supply to the fetus in a situation of increased oxygen demand [41].

In a previous study, we found that altered glucose metabolism during pregnancy is associated with changes in a particular HMO, i.e., 3′SL, in maternal serum [22]. In this study, we hypothesized that GDM is associated with changes in cord blood HMO concentration or composition. Their presence in the fetal circulation allows their interaction with endothelial cells lining the feto-placental vasculature with effects on feto-placental endothelial function. Therefore, we further hypothesized that potential GDM associated changes in the concentration of distinct HMOs influence angiogenesis. To study this, we used primary feto-placental endothelial cells (fpEC) as in vitro model.

## 2. Materials and Methods

### 2.1. Human Subjects

Pregnant women with normal glucose tolerance (NGT) or with GDM diagnosed by universal screening with a 75 g oral glucose tolerance test (OGTT) using IADPSG/WHO 2016 criteria were recruited at the Department of Obstetrics of the Medical University of Graz. Exclusion criteria were multiple pregnancy, more than three consecutive miscarriages, fetal anomalies, pre-gestational diabetes, pre-pregnancy hypertension, or preeclampsia/HELLP. The study complied with the Declaration of Helsinki guidelines as revised in the year 2000 and was approved by the ethical review board of the Medical University of Graz (EK# 28-604 ex 15/16). All subjects provided written informed consent. Maternal age, anthropometrics, birth mode, infant sex, infant weight and length, and gestational age at birth were recorded. Based on our previous results in cord blood, we calculated the samples size needed to detect an effect size of 0.3 in 3′SL, given the standard deviation stays in this range, to be 25 per group to reach a statistical power of 80%.

Venous blood samples from healthy pregnant women were drawn after admission at the hospital for delivery and before giving birth. Mixed blood samples from the umbilical cord were collected immediately after delivery of the placenta. After centrifugation at 3500× *g*, serum samples were stored at −80 °C until analysis.

Feto-placental endothelial cells fpECs were isolated from placentas delivered after NGT pregnancies (EK# 25-008 ex 12/13; EK#29-319 ex 16/17).

### 2.2. Human Milk Oligosaccharide Standards

The 2′-fucosyllactose (2′FL), 3-fucosyllactose (3FL), lacto-N-tetraose (LNT), lacto-N-neo-tetraose (LNnT), lacto-N-fucopentaose (LNFP) 1, 2, and 3, lacto-N-difucohexaose 1 (LNDFH1), lacto-N-hexaose (LNH), and linear B6 trisaccharide (internal standard) were purchased from Dextra Laboratories, Reading, UK. Lactodifucotetraose (LDFT), 3′-sialyllactose (3′SL), 6′-sialyllactose (6′SL), 3′-sialyllactosamine (3′SLN), 6′-sialyllactosamine (6′SLN) and sialyl-lacto-N-tetraose (LST) a, b and c, and disialyl-lacto-N-tetraose (DSLNT) (Glycoset II) were purchased from Prozyme, Hayward, CA, USA.

### 2.3. HMO Isolation and Analysis by HPLC

Oligosaccharides were isolated from serum samples, as previously reported [22,25]. In brief, 50 µL serum was added to linear B6 trisaccharide solution as internal standard and subjected to chloroform methanol extraction followed by SPE using C18 columns and graphitized carbon columns. Isolated HMOs were fluorescently labelled with 2-aminobenzamide (2AB), as previously described [42]. The 2AB-glycans were separated by HPLC (Agilent 1100 instrument) with fluorescence detection on an TSKgel amide-80 column (inner diameter 3.0 mm, length 15 cm, particle size 3 µm; Tosoh Bioscience; Tokyo, Japan), with a linear gradient of a 50 mM-ammonium formate/acetonitrile buffer system. Separation was performed at 35 °C and monitored by a fluorescence detector at 360 nm excitation and 425 nm emission. Standard retention times were used to annotate HPLC peaks.

### 2.4. Pooled HMO Preparations and Control Compounds Used in Functional Assays

HMOs were isolated from pooled human milk as described elsewhere [42] and used in concentration of 100 µg/mL unless otherwise stated. The same preparation was used for all experiments. The 3′SL (Prozyme) was used in 30 µg/mL. HMOs from cord blood for the use in Matrigel assay were isolated from 5 cord blood sera (NGT pregnancies) according to a protocol previously published with slight modification [43]. The 1 mL serum was diluted 1:1 with water and centrifuged at 6800× *g* for 15 min to remove lipids. A total of 500 µL of delipidated fractions were added to 1 mL chloroform-methanol (2:1) in a microtube and centrifuged at 1300× *g* for 15 min. Chloroform-methanol extraction was repeated, 300 µL water was added to the aqueous phase which was again centrifuged to pellet proteins. Supernatants were further deproteinated with C18 columns (500 mg/2.8 mL; Thermo Fisher, Waltham, MA, USA) and desalted using carbograph columns (25 mg/1 mL; Thermo Fisher). Isolated and dried cord blood HMOs were pooled and brought up to the original blood volume (10 mL) with medium to mimic physiologic concentrations. The same preparation was used for all experiments. Unless otherwise stated, HMOs used in the in vitro experiments are derived from pooled human milk for reasons of feasibility (limited volume of serum samples) and consistency (same HMO composition for all experiments).

As positive control, a mixture of the proangiogenic factors tumor necrosis factor (TNF)-α (10 ng/mL; Reliatech, Wolfenbüttel, Germany), vascular endothelial growth factor (VEGF, 650 pM; Sigma Aldrich, St. Louis, MO, USA) and fibroblast growth factor (FGF)-2 (10 ng/mL; Sigma Aldrich), abbreviated TVF, was used in functional assays. As a non-HMO glycan control, D-lactose monohydrate (Sigma Aldrich) in equimolar concentration (17 µg/mL) to 3′SL (30 µg/mL) was used.

### 2.5. Endotoxin Removal

HMOs isolated from human milk as well as commercially produced HMOs are known to contain endotoxin. To exclude effects of endotoxin in in vitro experiments, we removed endotoxin using polymixin B [44] by High Capacity Endotoxin Removal Resin (Thermo Scientific, Rockford, IL, USA) according to manufacturer’s instructions.

### 2.6. Feto-Placental Endothelial Cells

Primary arterial feto-placental endothelial cells (fpECs) were isolated from 10 individual placentas following a standard protocol [45]. Briefly, chorionic arteries were dissected, and endothelial cells isolated by perfusion with a collagenase/dispase (Roche, Mannheim, Germany) solution. Cells were resuspended in endothelial basal medium (EBM, Lonza, Walkersville, MD, USA) supplemented with the EGM-MV BulletKit (Lonza, Walkersville, MD, USA) on 1% (*v*/*v*) gelatin-coated flasks. Isolated fpECs were cultured at 37 °C and 12% oxygen, and used up to passage 10. Cells were characterized by immunocytochemical analysis with positive staining for the endothelial cell markers VWF (von Willebrand factor) and CD31, and negative for the SMA (smooth muscle actin) and the fibroblast marker CD90.

### 2.7. Proliferation

To investigate HMO effects on the proliferation rate of fpECs, we analyzed the metabolic activity and cell viability by colorimetric MTT assay (In Vitro Toxicology Assay Kit, MTT Based; Sigma Aldrich, St. Louis, MO, USA), according to manufacturer’s instructions. The 2.5 × 10^4^ and 5 × 10^4^ cells were seeded per well of a 96-well-plate. Triplicates were either untreated or treated with TVF (as positive control), HMOs, 3′SL, or lactose (as non-HMOs glycan control). The cells were incubated at 37 °C and 12% oxygen for 24, 48 or 96 h. Optical density (OD) at 570 nm was measured using a microplate reader.

### 2.8. Immunocytochemistry Staining for Phalloidin

FpECs (5 × 10^4^ cells/well) were seeded onto 1% gelatine coated chamber slides, cultured for 24 h and treated with either 5% FCS supplemented M199 medium alone, or medium containing TVF, pooled HMOs, 3′SL, or lactose for 24 or 48 h. Monolayers were washed with HBSS 1× (Gibco), air dried and fixed with 3.7% formaldehyde solution in PBS for 10 min at room temperature. After washing with PBS, cells were permeabilized with Triton X-100 in PBS, and again washed with PBS. After blocking with 2% BSA in PBS for 30 min, slides were incubated with Mouse Vinculin primary antibody (Neo Markers, Lab Vision; diluted 1:50) for 90 min at room temperature. Stained cells were washed thrice and simultaneously incubated with DL650 goat anti mouse (diluted 1:100, Thermo Scientific) as secondary antibody for vinculin staining and Phalloidin-488 FITC (1:20, Alexa, Thermo Scientific) for F-actin staining. After washing with PBS, Pro Long Gold Antifade DAPI (4′,6-Diamidin-2-phenylindol) mounting medium (Life Technologies, Carlsbad, CA, USA) was used to stain nuclei. After overnight drying, fluorescent staining was observed using a Zeiss LSM 510 META scanning laser confocal microscope. Integrated lasers are UV 405 nm (for violet excitation of dyes such as DAPI), tuneable Argon 458/477/488/514 nm (458, 477, and 488 for blue excitation of dyes such as FITC) and Helium-Neon 633 nm (for red excitation dyes, such as vinculin secondary antibody DL650 goat anti mouse). Zeiss LSM 510 software was used to observe F-actin organization.

### 2.9. The 2D In Vitro Network Formation Assay

The highly reproducible 2D Matrigel assay determines cell migration and network formation [46]. Using this assay, we investigated whether HMOs affect network formation in fpECs, and identified the concentrations with the most pronounced effects. FpECs were seeded with supplemented EBM in 12% O_2_, at 37 °C, and grown for 24 h. Cells were incubated for 4 to 24 h with HMOs (isolated from pooled human milk or pooled cord blood from NGT pregnancies as described above) in non-supplemented EBM containing 5% FCS. FpECs were harvested with Accutase (PAA, Innovative Cell Technologies, San Diego, CA, USA), resuspended in non-supplemented EBM containing 5% FCS. 10^4^ viable cells/well were seeded in triplicates on 96-well plates pre-coated with 50 µL of growth factor-reduced Matrigel (Corning, Bedford, MA, USA). Images of the 2D network formation were taken hourly by Cell IQ (CM Technologies Oy, Tampere, Finland). Total length of networks formed after 12 h was quantified using the AngioJ-Matrigel assay plugin for the ImageJ software (NIH).

### 2.10. The 3D Spheroid Sprouting Assay

Spheroids for the 3D spheroid sprouting assay were generated according to the hanging drop cell culture protocol [47]. FpECs were detached and mixed with methylcellulose solution (1 × 10^3^ cells per 25 μL). Drops (25 µL each) of the cell suspension were allocated on the lid of a non-adherent plastic dish, which was inverted and incubated for 24 h at 21% O_2_ and 37 °C. After visual evaluation of spheroid formation under the microscope, spheroids were harvested with HBSS, and the pooled suspension was centrifuged 5 min at 300× *g* to pellet the spheroids. On ice, the spheroids were overlaid with 322 μL methylcellulose stock solution containing 40% FCS. Subsequently, 248 μL NaHCO_3_ was added, and the solution was gently mixed with 1100 μL Type 1 Collagen to reach a final concentration of 2 mg/mL. After addition of 17 μL NaOH, the collagen-spheroid solution was mixed and transferred to 3 wells of a 24-well plate (500 μL/well). The plate was incubated at 37 °C, 21% O_2_ for two hours to allow collagen to polymerize. Then, complete M199 medium (Lonza, Basel, Switzerland) supplemented with 10% human serum (Atlanta Biologicals) and 10% new born calf serum (NBCS, Thermo Fisher Scientific) containing pooled HMOs, 3′SL or control compounds (TVF, lactose) was added and incubated for a maximum of 16 h. To terminate the experiments, the media were aspirated, and spheroids were fixed for two hours with 500 μL warm 4% formaldehyde. After aspirating the formaldehyde, wells were filled with 800 μL PBS, sealed with Parafilm and stored at 4 °C until imaging with a Cell-IQ V2 MFL system (Chip-Man Technologies Ltd., Tampere, Finland) [46].

### 2.11. Fibrin Angiogenesis Assay

To investigate HMO effects on in vitro tube formation, 3D fibrin tube formation assays were performed [48,49]. Fibrinogen (4 mg/mL) CoaChrom Diagnostica, Maria Enzersdorf, Austria) was dissolved in preheated M199 medium avoiding premature matrix coagulation. After 60 min at 37 °C, the solution was filtered through a 0.45 μm filter and kept on ice. Thrombin IIa (0.03 U/mL, CoaChrom Diagnostica) was added, and the solution was filled into a 96-well plate. After one hour at room temperature plates were put at 37 °C for one hour to allow fibrin polymerization. A total 100 μL of CM199 medium with 5% FCS was added to each well, and plates were incubated for two hours at 37 °C to inactivate thrombin IIa. Then, fpECs (3.5 × 10^4^ cells per well) were seeded onto the fibrin matrix. After overnight adhesion of cells, plates were tapped to loosen dead cells, medium was removed, and 100 μL medium containing pooled HMOs, 3′SL or control compounds was added to the wells. Medium was replaced by fresh treatment medium every two to three days until tube formation was visually observed. For assay termination, cells were washed and fixed in warm 4% formaldehyde for two hours. Then, formaldehyde was removed and 200 μL PBS was added to each well, sealed with Parafilm and stored at 4 °C until imaging with Cell-IQ V2 MFL system.

### 2.12. Statistical Analysis

Data are presented as means and standard deviation (SD) for normally distributed data and as medians and interquartile ranges (IQR) for skewed data such as HMO concentrations. Differences in maternal characteristics and HMO concentrations between GDM and NGT pregnancies were tested using the Mann–Whitney-U-test. Linear regression analysis was performed to assess associations between maternal and infant characteristics and cord blood HMO concentrations. Effect of treatment in functional assays with either HMOs, 3′SL, or lactose compared to no-treatment control were analyzed by repeated measurements one-way ANOVA with multiple comparison testing. Statistical analyses were performed using SPSS (version 25) (IBM SPSS, Chicago, IL, USA), graphs were plotted in GraphPad Prism (version 9.00; GraphPad Software. La Jolla, CA, USA). Statistical significance was assumed when *p* < 0.05.

## 3. Results

### 3.1. HMOs in Cord Blood Are Altered in GDM

#### 3.1.1. Cohort Characteristics

Table 1 shows subject characteristics from normal glucose tolerance (NGT) control (*n* = 25) and GDM (*n* = 26) pregnancies. Women with GDM had either received dietary recommendations alone (*n* = 13) or additional insulin or metformin treatment (*n* = 13). GDM and NGT samples were matched for pre-pregnancy BMI and delivery mode. BMI at delivery, gestational age at birth, infant sex and infant anthropometrics were not significantly different in the two groups. However, maternal age was significantly higher in women who developed GDM (33.3 ± 5.5 years) compared to women with NGT pregnancies (28.5 ± 4.5 years), and placenta weight was significantly lower in GDM pregnancies.

#### 3.1.2. The 3′SL and 3′SLN Increased in GDM Cord Blood

**Figure 1:**
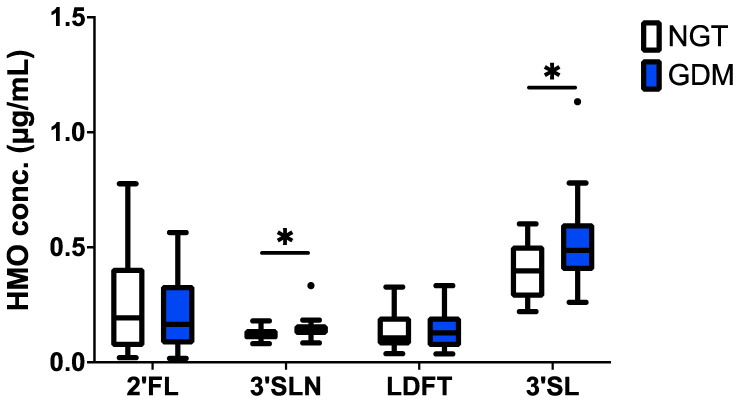
HMO concentrations in cord blood serum are different after GDM pregnancy. Tukey box-and-whiskers plot shows 2′-fucosyllactose (2′FL), 3′-siallylactosamine (3′SLN), lacto-difucotetraose (LDFT) and 3’-sialyllactose (3′SL) concentrations in cord blood from pregnancies with normal glucose tolerance (NGT, open boxes) and GDM (blue boxes). NGT control *n* = 25, GDM *n* = 26; significance was determined by the Mann–Whitney-U-test; * *p* < 0.05.

HMO analysis by HPLC demonstrated the presence of up to 16 HMOs (2′FL, 3FL, LDFT, 3′SL, 6′SL, LNT, LNnT, LNFP1, 2, 3, LSTa, b, c, LNDFH, LNH, DSLNT) and two trisaccharides featuring a lactosamine instead of the lactose backbone (3′SLN and 6′SLN), all consistently found in maternal serum and cord blood [21,22,33]. Total HMO concentration was not different between GDM and NGT controls (data not shown). However, among the four most abundant individual HMOs, 2′FL, 3′SLN, LDFT and 3′SL, concentrations of the sialylated 3′SL and 3′SLN were significantly higher in cord blood samples from GDM pregnancies compared to NGT controls (median 0.77 vs. 0.63 nmol/mL and 0.20 and 0.17 nmol/mL, Mann–Whitney-U-Test, *p* = 0.02) (Figure 1).

#### 3.1.3. Maternal Factors Are Associated with 3′SL in Cord Blood

Linear regression analysis was used to estimate the association of maternal characteristics (age, height, pre-pregnancy weight, pre-pregnancy BMI, weight at delivery, BMI at delivery, parity) and metabolism (blood glucose at oGTT and GDM status [yes/no]) on cord blood HMOs. In addition, associations of parity and mode of delivery (primary C-section [yes/no]) and infant characteristics (gestational age at birth, birth weight, sex) with HMOs were assessed (Table S1). In univariate analysis, maternal GDM diagnosis was associated with 3′SL and 3′SLN in cord blood (beta 0.17, 95%CI 0.03 to 0,31, *p* = 0.02; and beta 0.03, 95%CI 0.00 to 0.06, *p*= 0.05). Maternal height and weight, BMI, age, or parity were not associated with the concentration of 3′SL or 3′SLN in cord blood, nor were infant gestational age at birth, birth weight or sex. We found, however, primary C-section to be associated with lower cord blood 3′SL (beta −0.24, 95%CI −0.38 to −0.10, *p* = 0.001). After adjustment for primary C-section, GDM (beta 0.13, 95%CI 0.01 to 0.24, *p* = 0.04) was still significantly associated with cord blood 3′SL (beta 0.5, 95%CI 0.02 to 0.27, *p* = 0.04), but not with 3′SLN (beta 0.03, 95%CI −0.002 to 0.06, *p* = 0.07). A sub-analysis of GDM pregnancies showed no association of drug treatment (i.e., insulin or metformin [yes/no] in addition to diet) on 3′SL when corrected for mode of delivery (data not shown).

Depending on the presence of 2′FL in cord blood, we assigned the women’s secretor status which was comparable in the NGT and GDM women. Out of the 25 cord blood samples of the NGT controls, 5 were assigned a negative secretor status (24%), out of the 26 GDM samples, and 6 were secretor negative (23%). We excluded non-secretor samples (*n*= 11), and analyzed the groups again for differences in HMOs. The results for 2′FL and LDFT in the secretor group were not different between GDM (*n* = 21) and NGT (*n* = 19) women. However, the difference in median concentration of 3′SL (0.73 vs. 0.56 nmol/mL; *p* = 0.007) and 3′SLN (0.21 vs. 0.17 nmol/mL, *p* = 0.04) was higher significant without non-secretors (multiple Mann–Whitney-U-test). Both GDM diagnosis and primary C-section were associated with cord blood 3′SL (beta 0.10, 95%CI 0.03 to 0.18, *p* = 0.01 and beta −0.12, 95%CI −0.20 to −0.03, *p* = 0.01, respectively) in secretor positive women. Both variables were also associated with 3′SLN concentrations in cord blood (beta 0.02, 95%CI 0.00 to 0.04, *p* = 0.046 for GDM and beta −0.02, 95%CI −0.04 to −0.003, *p* = 0.03 for primary C-section) in this subgroup.

The observed concentration differences in 3′SL and 3′SLN between GDM and NGT pregnancies prompted studies of whether HMOs can influence endothelial function and angiogenesis in vitro. To this end, we specifically investigated effects of HMOs on cytoskeletal reorganization, migration, proliferation, sprouting and tube formation in primary feto-placental endothelial cells (fpECs).

### 3.2. HMOs Affect In Vitro Angiogenesis of fpECs

#### 3.2.1. HMOs Stimulate Network Formation of fpECs

**Figure 2:**
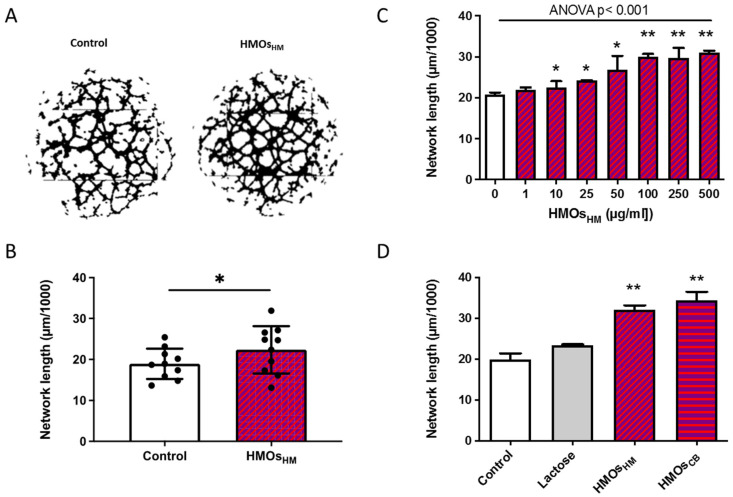
Human milk oligosaccharides (HMOs) dose-dependently increased network formation in primary fpECs. fpECs were pre-incubated for 24 h with EBM medium supplemented with 5% FCS with or without HMOs prior to use in a 2D network formation assay (Matrigel). (**A**) Representative images of network formation after 24 h treatment with (right) or without (left) pooled HMOsHM prior to use in a 2D network formation assay. (**B**) Bar plot shows total tube length at 12 h after seeding. A set of *n* = 10 fpEC isolations from healthy placentas was used, each in 2 to 3 independent experiments in technical triplicates (mean ± SD, *n* = 10, paired *T*-test). (**C**) fpECs were treated with serial concentrations (0–500 µg/mL) of pooled HMOsHM and incubated for 24 h showing a dose-dependent effect on 2D network formation. Two independent experiments were performed with two different fpEC isolations in triplicates (means ± SEM, ANOVA with multiple comparison). (**D**) Network formation was similarly increased when fpECs were incubated with HMOs isolated from pooled human milk (HMOsHM) as from pooled cord blood (HMOsCB red- purple vertically striped) and significantly different to no treatment and non-HMO control for 24 h. Two independent experiments were performed with two different fpEC isolations in triplicates; means ± SEM, paired *t*-test shows significance difference vs. control (white bar). * *p* < 0.05; ** *p* < 0.01. CB, cord blood; HM, human milk.

Using the Matrigel assay, HMOs isolated from pooled human milk (100 µg/mL) significantly increased network formation in fpECs (Figure 2A,B). In a next step, we tested different HMO concentrations to identify the effective concentration range. To this end, from the previous experiment, we selected two fpEC isolations with a pronounced response to HMOs_HM_ (Figure 2B). Concentrations from 5 µg/mL to 500 µg/mL dose-dependently increased network formation, reflected by an increase in total tube length in the Matrigel assay (Figure 2C). The magnitude of the effect reached a plateau at 100 µg/mL. Hence, this HMO concentration was chosen for all further experiments. We had previously shown that HMO profiles in cord blood serum share major oligosaccharides with HMO isolations from breast milk [33]. Therefore, we next investigated whether cord blood HMOs had comparable effects on fpECs to HMOs isolated from human milk. HMOs were isolated from pooled cord blood of 5 neonates obtained after normal pregnancies and taken up in culture medium mimicking physiological concentrations. Cord blood HMOs increased network formation of fpECs similar to the effect of 100 µg/mL HMOs isolated from human milk (41.3% vs. 36.26%, respectively) (Figure 2D). For feasibility reasons, because HMO concentrations in human milk exceed those in cord blood by several orders of magnitude, we used HMOs isolated from human milk for the following experiments.

#### 3.2.2. HMOs Increase Proliferation of fpECs

**Figure 3:**
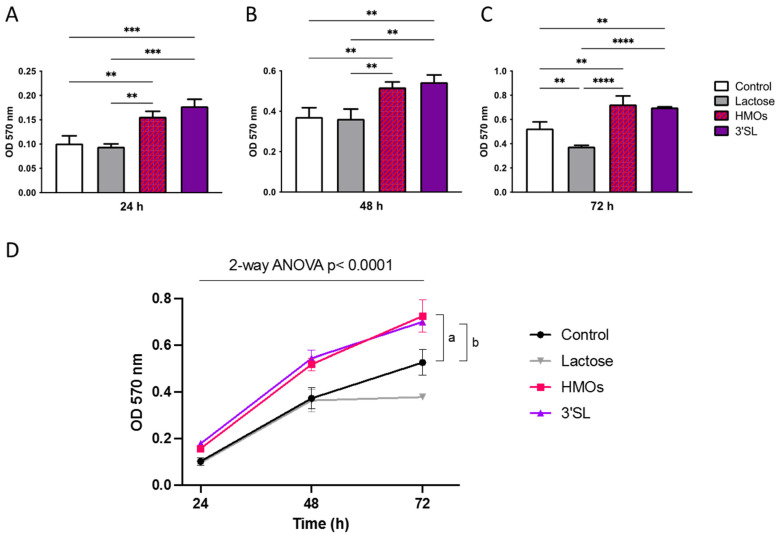
Human milk oligosaccharides (HMOs) increased proliferation of fpECs. The 5 × 10^4^ fpECs were resuspended in medium with and without HMOs, 3′SL or lactose, and seeded into wells of a 96-well-plate. MTT dye was added at the indicated time points, and fpECs were incubated for 4 h before the reaction was terminated. Cell viability was determined by measuring the optical density (OD) at 570 nm in an enzyme-linked-immunosorbent assay (ELISA) microplate reader after (**A**) 24, (**B**) 48 and (**C**) 72 h post seeding. Asterisks indicate significant differences between treatments; ** *p* < 0.01, *** *p* < 0.001 and **** *p* < 0.0001. (**D**) Time course of OD at the above shown individual time points (24, 48 and 72 h). Values shown are the mean ± SEM of three different fpEC isolations, each seeded in triplicates. Two-way ANOVA with two-stage Benjamini, Krieger and Yekutieli FDR procedure; the letter a indicates significant difference of HMOs vs. control; the letter b indicates significant difference of 3′SL vs. control.

As a measure for proliferation, we used a MTT based assay to investigate effects of HMOs and 3′SL alone 24, 48 and 72 h after seeding. Treatment of fpEC with HMOs (100 μg/mL) and 3′SL (30 μg/mL) showed a comparable effect size, which was similar to that of TVF (a combination of TNF-α, VEGF and FGF-2, used as positive control; data not shown), and increased proliferation at each time point. Lactose, which was used as a non-HMO glycan control, had no effect on proliferation (Figure 3).

#### 3.2.3. HMOs Alter Cytoskeleton Organization in fpECs

**Figure 4:**
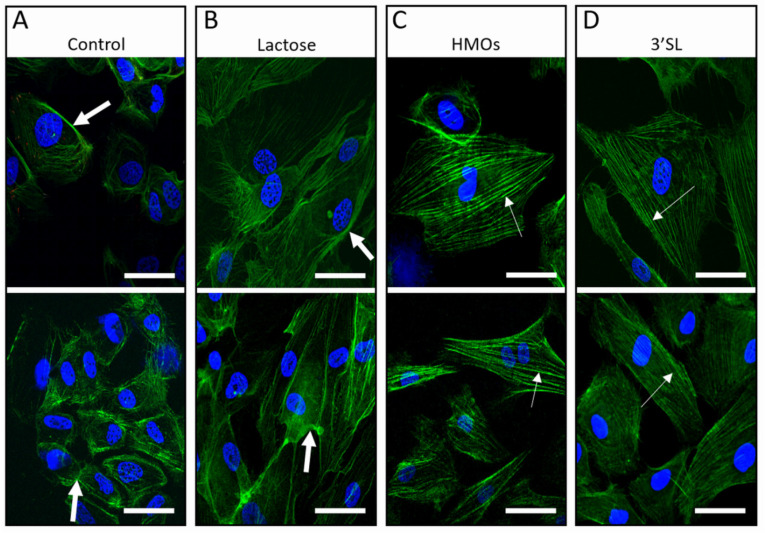
Effect of HMOs on actin cytoskeleton organization of cultured fpECs. The 5 × 10^4^ cells per well were seeded on gelatin coated chamber slides. fpECs were cultured for 24 h in M199 medium containing 10% hPS and 10% nBCS followed by a treatment of 24 with M199 medium containing HMOs, 3′SL or lactose, or left untreated (two representative images each). (**A**) Control conditions (untreated cells) and (**B**) cells treated with lactose as non-HMO showed membrane ruffle formation (big white arrows) and weak phalloidin staining. Treatment with (**C**) HMOs (**D**) 3′SL led to parallel, highly ordered and strongly stained stress fibers (thin white arrows), similar to the positive pro-angiogenic control with TVF (TNF-α, VEGF and FGF-2) (not shown). There was no difference between the 24- and 48-h treatments. Similar results were obtained in five individual experiments with different fpEC isolations (*n* = 5). 3′SL, 3′-sialyllactose; scale bar = 50 µm.

We next investigated HMOs effects on actin cytoskeleton visualized after 24 or 48 h of treatment by laser scanning confocal microscopy. Under control conditions, i.e., medium containing FCS and growth supplements, there was no difference in cytoskeleton organization observed between the 24 and 48 h time point. Untreated control cells, grown under starving conditions, i.e., in medium containing 5% FCS but without growth supplements, showed membrane ruffling (big white arrows) and weakly pronounced F-actin fibers (Figure 4A). The cells appeared faint with a contracted, shrunken shape, compared to their original round shape. Cells were polygonal and had wispy actin filaments, a characteristic of serum-starved cells. Their actin filament bundles were markedly shorter and not longitudinally aligned with a disorganized appearance. Plasma membranes were only weakly stained with phalloidin, similar to treatment with lactose, used as negative, non-HMO glycan control (Figure 4B). Stimulation with TVF served as a positive control, inducing actin reorganization and migration [50]. fpECs treated with HMOs or 3′SL underwent profound changes in shape and actin/cytoskeleton organization (Figure 4C,D). F-actin stress fibers stretched through the cell body in a parallel and organized way (thin white arrows, Figure 4C,D). No membrane ruffles were noticeable, and the cells presented an intense F-actin fiber staining.

#### 3.2.4. HMOs Stimulate In Vitro Angiogenesis of fpECs

To study the effect of HMOs on 3D in vitro angiogenesis of fpECs, we employed two assays: spheroid sprouting assay was used to analyze the effect of HMOs on sprouting of fpECs within a gelatin matrix, and fibrin tube formation assay was used to study the formation of luminal tubes within a fibrin matrix.

**Figure 5:**
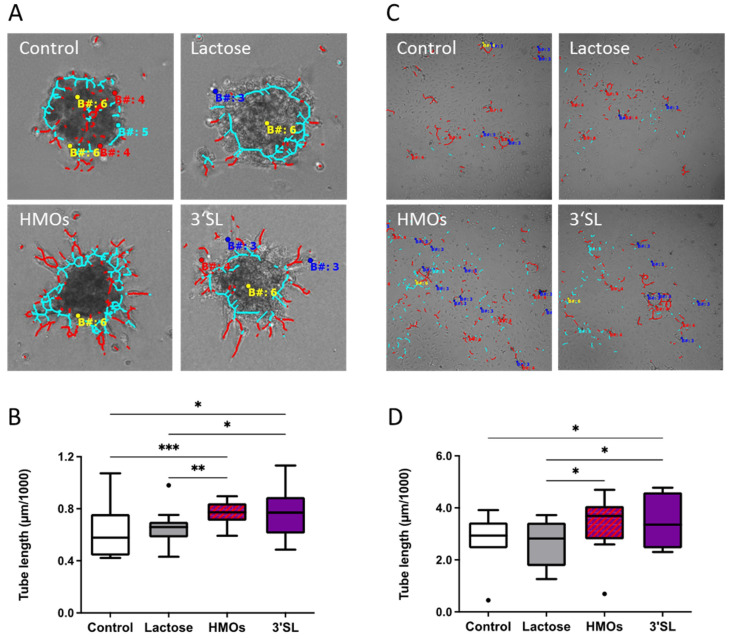
HMOs stimulated in vitro angiogenesis of fpECs. A set of *n* = 10 individual fpEC isolations from healthy placentas were used for spheroid assays (**A**,**B**) and fibrin assays (**C**,**D**). (**A**,**B**) Spheroid assay showed significantly increased sprouting in the presence of HMOs (100 μg/mL) or 3′SL (30 μg/mL) as compared to untreated control. Lactose served as non-HMO control. (**A**) Representative image of spheroids analyzed with Cell-IQ analyzer software. Red lines are measuring the sprouts. (**B**) Box blots show median of tube length in the respective treatments. (**C**) Representative images of tubes analyzed with the Cell-IQ analyzer. The red dots and lines represent the tubes. (**D**) Fibrin assay showed a significant increase in total tube length upon treatment with HMOs (100 μg/mL) and 3′SL (30 μg/mL), compared to untreated cells and cells treated with lactose. Repeated measures ANOVA with uncorrected Fisher’s LSD; * *p* < 0.05; ** *p* < 0.01; *** *p* < 0.001; *n* = 10.

Sprouting was significantly increased in the presence of HMOs (100 μg/mL) or 3′SL (30 μg/mL) as compared to untreated control (Figure 5A,B). Lactose served as non-HMO control and TVF as positive control.

The fibrin assay showed a 24.3% and 25.3% increase in total tube length upon treatment with HMOs and 3′SL, respectively, compared to untreated cells. Lactose had no significant effect on the growth of vascular structures into the fibrin matrix (Figure 5C,D).

## 4. Discussion

Our findings not only confirmed the presence of HMOs in cord blood, which we previously demonstrated in a different cohort [33], but found two novel, key results: (1) The cord blood concentration of 3′SL as well as its glucosamine analog, 3′SLN, was increased in pregnancies complicated with GDM. This suggests that fetal HMO concentrations can be modulated by maternal metabolism. (2) HMOs isolated from human milk or cord blood, as well as the single HMO 3′SL had profound effects on fpECs in vitro. They increased proliferation, changed cytoskeleton organization and induced tube formation; all processes critically involved in angiogenesis. Provided that these in vitro findings reflect in vivo effects, HMOs present in the fetal circulation can have an impact on feto-placental endothelial angiogenesis.

The 3′SL, one of the most prominent HMOs in cord blood, and the lower abundant 3′SLN were increased in pregnancies complicated with GDM, demonstrating an influence of maternal glucose metabolism on fetal HMO composition. Further, 3′SLN is found in blood and urine, and its concentrations are highly correlated with those of 3′SL [21,22,33], indicating a co-regulation of 3′SL and 3′SLN. In our previous study in a Dutch pregnancy cohort of obese and overweight women [22], 3′SL and 3′SLN concentration in maternal serum were significantly higher in women who later developed GDM. Our present results indicate that GDM associated alterations of maternal HMOs are also found in the neonatal circulation albeit at different time periods of pregnancy. In maternal blood, elevated 3′SL was detectable only at gestational Weeks 14 and 22, but not at Week 35. Why the GDM effect on maternal serum 3′SL resolved in late pregnancy remains unclear. The 3′SL, as well as 3′SLN, is not correlated between maternal and fetal circulation [33], suggesting complex transport mechanisms across the placental barrier, placental or fetal sequestration or all of those. Longitudinal studies measuring HMOs in maternal blood samples throughout pregnancy paired with cord blood samples are needed to fully understand this observed discrepancy. Furthermore, underlying reasons driving changes in 3′SL in GDM still remains to be elucidated.

Interestingly, we also found an effect of delivery mode, i.e., primary C-section, on 3′SL concentration in cord blood (Table S1). Higher 3′SL was measured in cord blood after mothers had experienced labor, delivering their infants by spontaneous vaginal birth, secondary C-section or other assisted methods, as compared to primary C-sections in the absence of labor. Labor is a state of physiological inflammation, and onset of myometrium contractions is directly related to profound changes in hormones and cytokine signaling. In a previous study, we found higher maternal serum 3′SL to be associated with sterile inflammation associated with preterm labor and preterm birth [25]. Inflammatory conditions have been associated with higher sialylation of both cell-surface and secreted glycans [51] and, thus, inflammation itself might be associated with higher serum 3′SL. In the total cohort, the absence of labor, i.e., primary C-section, was associated with lower 3′SL. However, after adjustment for primary C-section, the association of GDM with higher 3′SL remained significant (supplementary Table S1). Future studies need to include cord blood glucose and insulin parameters as well as inflammatory markers to allow analyses of potential associations of fetal metabolism with cord blood HMOs.

Since HMOs in cord blood contact the feto-placental endothelium, we investigated the effect of HMOs and 3′SL on angiogenesis in fpEC. Our results strongly suggest a novel function of HMOs in angiogenesis that might be modulated by maternal metabolic derangements such as GDM. Previous microarray analysis showed that potential HMO receptors such as Galectins, C-lectins, TLR-4 or EGFR are highly expressed in fpEC (accessible through GEO Series accession number GSE44368) [52]. These putative HMO interaction partners have been shown to play a role in angiogenesis [53,54,55]. As HMOs are present in fetal blood circulating through feto-placental vessels and capillaries, isolated primary fpECs represent an ideal model to study effects of HMOs on physiologic angiogenesis in the feto-placental vasculature. Angiogenesis encompasses several distinct processes that include migration, cytoskeleton rearrangement, proliferation, sprouting and tube formation. We have used various assays to study these individual processes. We showed that HMOs dose-dependently increased migration, reflected by network formation of fpECs on Matrigel. HMOs or 3′SL alone strongly promoted proliferation of fpECs, and altered actin organization with induction of stress fibers, a process indicating the activation of cells which precedes angiogenesis [50,56]. HMOs and 3′SL significantly increased in vitro angiogenesis in two independent 3D angiogenesis assays, i.e., spheroid sprouting assay (32%) and fibrin tube formation assay (24%), which assesses the formation of luminal structures within a matrix [57]. Together our findings strongly argue for a novel role of HMOs in modulating feto-placental angiogenesis and thus, placental and fetal development.

Few studies have investigated effects of some individual HMOs on (pathophysiologic) angiogenesis with similar, but also seemingly contradicting findings [58]. Specifically, LNnT showed pro-angiogenic properties in the context of wound healing [59,60], and 2′FL induced directed migration in human microvascular endothelial cells (HMVECs), and promoted in vitro and in vivo angiogenesis [61,62]. These studies corroborate our findings that HMOs can induce pro-angiogenic signaling in endothelial cells. The HMO preparation used in our experiments is structurally complex and comprises fucosylated HMOs, such as 2′FL, unmodified HMOs such as LNnT, and sialylated structures such as 3′SL [42]. All these HMOs are also detectable in cord blood [33] rendering this HMO preparation an adequate approximation of the physiologic composition of HMOs expected in the feto-placental vasculature. Of note, none of the previous studies removed endotoxin, which is a common contaminant due to production process of commercially available HMO structures [63]. We also showed that 3′SL alone, a prominent HMO in cord blood profiles and increased by GDM, exerts pro-angiogenic effects on fpEC. Further, 3′SL is also known to bind to Galectin-3 [14] and Galectin-9 [64], factors proposed to be critically involved in placental angiogenesis [53]. Contrasting our results, another recent study reported anti-angiogenic properties of 3′SL, inhibiting VEGFR-2 activation and, thus, tumor progression [65]. These differences in direction of the effect might be explained firstly, by the use of endothelial cells from different vascular beds (fpEC vs. HMVECs, HUVECs) and secondly, by the distinct experimental designs, i.e., direct treatment with HMOs vs. treatment with HMOs after direct or indirect stimulation with pro-angiogenic factors. The latter aimed to investigate 3′SL as potential candidate to antagonize VEGFR2 mediated pathophysiologic neovascularization in cancer [65,66]. We added HMOs directly onto primary fpECs without prior angiogenic stimulation. In a specific pathological pro-angiogenic environment, individual HMO structures might have differently directed or pronounced effects on angiogenesis depending on endothelial phenotype. In this line of thoughts, HMOs might contribute to modulating physiologic feto-placental angiogenesis; in GDM, HMOs might then adapt feto-placental angiogenesis to adequately respond to fetal needs. It will also be interesting to investigate the effect of HMOs on fpECs isolated from GDM placentas to see whether the response to HMOs is different in diabetic vs. normal fpECs. Moreover, it is tempting to speculate that 3′SL and other HMOs present in the maternal and fetal circulation play a role in pregnancy and fetal/neonatal development, fine tuning and supporting angiogenesis that is physiologically increased in these phases of intensive tissue remodeling [41,67]. In the future, we aim to investigate underlying mechanisms of the HMO mediated effect on in vitro angiogenesis.

Strengths and limitations. One of the strengths of this work is the use of primary fpECs allowing to study HMOs as relevant pregnancy and lactation specific modulators of physiological angiogenesis in pregnancy, and also consider HMO effects in the context of metabolic deranged environment. Another strength of this study is the use of different 2D and 3D angiogenesis assays to account for the different aspects of angiogenesis. However, it should be mentioned that the 3′SL concentration (30 µg/mL) used in the in vitro experiments exceeded concentrations found in cord blood by approximately 60×. While it is clear that typically, in vitro assays only incompletely reflect the in vivo conditions, not accounting for e.g., flow or local concentrations and interaction, we chose this concentration for several reasons: (a) 3′SL represents about 30% of total HMOs in cord blood, and 100 µg/mL pooled HMOs showed a robust effect in the initial Matrigel assay. (b) Other in vitro studies in endothelial cells used similar concentrations for total or individual HMOs [11,59,65]. Future studies should investigate further details on HMO effects on in vitro angiogenesis dependent on concentration, exposure time, and HMO structure.

A limitation may be that, in an attempt to control for BMI, we had a relatively high proportion of obese women in the NGT control group. Maternal prepregnancy BMI did not have an independent effect on cord blood 3′SL when controlled for GDM and/or delivery mode. However, it would also be interesting to also have body fat mass measurements available to more specifically study effect of maternal obesity on fetal HMOs. Studies have shown that non-diabetic obese compared normal weight women show a greater risk for high HbA1c at delivery indicating dysglycemia in late pregnancy [68]. Thus, to ensure that women in the control group maintained glycemic homeostasis also later in pregnancy, we would have required maternal blood samples at delivery to measure HbA1c and other glucose/insulin parameters in all participants. Despite a potential reduction of the GDM effect as observed in the mothers, we could still detect a significant difference in 3′SL in GDM compared to NGT cord blood. Future studies need to obtain information on the metabolic status of the fetus. Another limitation was the relatively small proportion of spontaneous births in our cohort, 62% (NGT) and 72% (GDM) were C-sections, not allowing for separate analyses of spontaneous births and C-sections. However, we were able to account for the influence of labor on 3′SL.

## 5. Conclusions

In summary, this study confirmed the presence of HMOs in cord blood, and strongly suggests a GDM associated difference in HMOs in the fetal circulation. Our results further indicate that HMOs increase feto-placental angiogenesis in vitro via induction of migration, cytoskeleton rearrangement, proliferation, sprouting and tube formation. GDM mediated changes in cord blood HMOs may contribute to and fine-tune GDM associated hypervascularization of the placenta as means of fetal protection from metabolically-induced hypoxia [41].

## Figures and Tables

**Table 1:** Subject characteristics.

Maternal and Infant Characteristics	NGT Pregnancies *n* = 25		GDM Pregnancies *n* = 26		*p*
Maternal age (years)	28.5	4.5	33.3	5.5	0.001
Height (cm)	167.0	6.8	164.8	4.4	0.16
Weight pre-pregnancy (kg)	81.1	18.7	79.3	17.1	0.71
BMI pre-pregnancy (kg/m^2^)	28.9	5.8	29.2	6.2	0.88
BMI category pre-pregnancy (*n*, %)					
Normal weight (BMI < 25)	7	28%	8	30%	0.54
Overweight (BMI 25–30)	7	28%	4	15%
Obese (BMI > 30)	11	44%	14	54%
BMI at delivery (kg/m^2^)	33.5	5.5	31.7	5.5	0.31
oGTT 0 h (mg/dL)	84	4.5	98	10.9	<0.0001
oGTT 1 h (mg/dL)	131	26.6	167	41.8	0.0011
oGTT 2 h (mg/dL)	104	18.5	132	23.2	<0.0001
Gestational age at birth (days)	274.2	7.5	275.8	7.1	0.46
Infant weight (g)	3482	529	3414	400	0.61
Infant length (cm)	51.0	2.1	50.0	4.2	0.31
Ponderal index (kg/m^3^)	26.2	2.3	28.5	10.3	0.28
Placental weight (g)	662	107	589	118	0.027
Infant sex (*n* male; %)	16	64%	15	59%	0.78
Delivery mode (*n* primary C-section, %)	18	72%	16	62%	0.54

Mean ± SD or *n* (%).

## Supplementary Materials

The following are available online at https://www.mdpi.com/article/10.3390/nu13124257/s1, Table S1: associations of maternal and infant characteristics with 3′SL and 3′SLN.

## Abbreviations

HMO	Human milk oligosaccharide
2′FL	2′-Fucosyllactose
LDFT	lacto-difucotetraose
3′SL	3′-sialyllactose
3′SLN	3′-sialyllactosamine
6′SLN	6′-sialyllactosamine
LNT	lacto-N-tetraose
LNnT	lacto-N-neo-tetraose
LNFP	lacto-N-fucopentaose
LNDFH1	lacto-N-difucohexaose 1
LNH	lacto-N-hexaose
LST	sialyl-lacto-N-tetraose
DSLNT	disialyl-lacto-N-tetraose
fpEC	feto-placental endothelial cells
GDM	gestational diabetes mellitus
oGTT	oral glucose tolerance test
