# Supplementary material for: Human Milk Oligosaccharides in Cord Blood Are Altered in Gestational Diabetes and Stimulate Feto-Placental Angiogenesis In Vitro
Source: Nutrients. 2021 Nov 26;13(12):4257. doi: 10.3390/nu13124257 (PMC8705424; doi:10.3390/nu13124257)
Supplement: Supplementary file 1 [file nutrients-13-04257-s001.zip › nutrients-1460128-supplementary.pdf]

Table S1: Results of linear regression analysis for the association of maternal and infant characteristics with cord blood 3’SL and 3’SLN

Maternal & infant characteristics	Cord blood 3'SL				Cord blood 3'SLN			
	N	Beta	95% CI	P	N	Beta	95% CI	P
Univariate analysis								
Age at delivery, y	51	0.004	-0.01; 0.02	0.59	51	0.001	-0.002; 0.004	0.43
Height, cm	51	-0.01	-0.02; 0.005	0.24	51	-0.001	-0.004; 0.002	0.67
Weight pre-pregnancy, kg	51	0.000	-0.005; 0.004	0.83	51	-0.000	-0.001; 0.001	0.89
BMI pre-pregnancy, kg/m^2	51	0.001	-0.01; 0.01	0.92	51	0.000	-0.003; 0.003	0.94
Weight at delivery, kg	48	-0.001	-0.004; 0.003	0.68	48	0.000	-0.001; 0.001	0.75
BMI at delivery, kg/m^2	48	-0.001	-0.01; 0.01	0.85	48	-0.001	-0.003; 0.002	0.56
OGTT 0 min, mg/dl	48	0.002	-0.005; 0.01	0.61	48	0.000	-0.001; 0.002	0.74
OGTT 60 min, mg/dl	46	0.001	-0.001; 0.003	0.51	46	0.000	0.000; 0.000	0.96
OGTT 120 min, mg/dl	44	0.003	0.000; 0.006	0.07	44	0.000	0.000; 0.001	0.22
GDM, yes vs no	51	**0.17**	**0.03; 0.31**	**0.02**	51	**0.03**	**0.000; 0.06**	**0.05**
Parity, no. pregnancies	51	-0.05	-0.13; 0.03	0.24	51	0.002	-0.02; 0.02	0.84
Primary C-section, yes vs no	51	**-0.24**	**-0.38; -0.10**	**0.001**	51	-0.03	-0.06; 0.01	0.12
Gestational age at birth, days	51	0.008	-0.002; 0.02	0.11	51	0.000	-0.002; 0.003	0.69
Birth weight, kg	51	-0.009	-0.17; 0.15	0.91	51	0.01	-0.03; 0.05	0.57
Infant sex, female vs male	51	0.02	-0.13; 0.17	0.77	51	0.02	-0.02; 0.05	0.32
Multivariate analysis								
Primary C-section, yes vs no	51	**-0.22**	**-0.36; -0.09**	**0.002**	51	-0.02	-0.06; 0.01	0.16
GDM, yes vs no	51	**0.15**	**0.02; 0.27**	**0.04**	51	0.03	-0.002; 0.06	0.07
